# Peak expiratory flow in normal medical students in Maiduguri, Borno state, Nigeria

**Published:** 2012-07-16

**Authors:** Bukar Bakki, Ahmad Hammangabdo, Mohammed Abdullahi Talle, Segun Oluwole, Haruna Yusuph, Mohammed Bashir Alkali

**Affiliations:** 1 Department of Medicine, College of Medical Sciences, University of Maiduguri, P M B 1414, Maiduguri, Nigeria; 2 Department of Medicine, Federal Medical Centre, Gombe, Nigeria

**Keywords:** Pulmonary function test, peak expiratory flow, prediction formulae, medical students

## Abstract

**Background:**

The assessment of lung function is of considerable importance in the diagnosis of respiratory diseases, normal reference values need to be determined. The peak expiratory flow (PEF) is a simple, reproducible and easily affordable test of lung function which has been used in resource poor countries like Nigeria. A study PEF was carried out in medical students of the University of Maiduguri and the result was compared with various prediction equations calculated in other parts of Nigeria.

**Methods:**

It was a cross-sectional study involving 255 medical students. Data was collected between March and June 2010 using MicroPeak™ peak flow meter (Micromedical MEI 2A2 Kent) as the instrument.

**Results:**

There was a statistically significant difference between the measured PEF and the predicted values based on different formulae derived from the different parts of Nigeria. However, the values in females in this study was consistent with the one obtained by one investigator in the north western part of the country. PEF positively correlated with the measured anthropometric parameters and age.

**Conclusion:**

The result of the study showed that the prediction formulae of Njoku et al and Salisu et al may be used in the assessment of PEF of individuals in this environment; however, further studies with larger sample size may be needed.

## Background

Pulmonary function tests play an integral role in the diagnosis and management of patients with respiratory diseases. Peak expiratory flow (PEF), or maximal expiratory flow, is the highest flow achieved during a forced expiration starting from the level of maximum lung inflation [1, 2]. Peak flow is one of the means of objectively assessing and monitoring the airway function of patients with bronchial asthma. PEF has several advantages over other spirometric measurements including suitability for field work, simplicity of PEF manoeuvre, cheap, robust and portable devices; and feasibility in non specialized centres. It is a favoured tool in epidemiologic studies and has the properties of a good screening test [3].

There is variation in the normal predicted values of lung indices among various ethnic groups [4, 5]. PEF is affected by anthropometric factors such as height, weight, chest circumference and body surface area [6] and these tend to be different among ethnic groups. Some studies on PEF have been carried out in both Nigerian children [7, 8] and adults [4, 6, 9]. These studies have been limited largely to the south western part of the country with predominantly one ethnic group and so national variation has not reported. There is little information from other areas, including the North East. We determined the PEF of a mixed group of 255 medical students and compared the results with the various prediction equations calculated in other parts of Nigeria.

## Methods

### Study population and sampling

Study participants were recruited using simple random sampling from the student population of the College of Medical Sciences University of Maiduguri to a cross-sectional survey. Data were collected between March and June 2010. Registered students aged 18–37 years were included in the study. Exclusion criteria included inability or unwillingness to take part in the study; self-reported history of significant cardiopulmonary diseases like acute myocardial infarction, pulmonary tuberculosis etc; and being a current smoker.

### Measurements

Height was measured without shoes to within 0.1 cm using a stadiometer. Weight was measured in kilograms to the nearest 0.5 kg with subjects wearing light clothing using a portable bathroom weighing scale (HANA BR-9012).

PEF was measured with a MicroPeak™ peak flow meter (MicroMedical MEI 2AZ Kent UK). All tests were performed with the subjects comfortably seated. Subjects were required to exhale as quickly as possible into the peak flow meter following maximum inspiration. Blowing technique was closely watched to ensure that a tight seal was maintained between lips and the mouthpiece of the peak flow meter. Three PEF manoeuvres were made by each subject, and the highest blow into each instrument was recorded. All measurements were carried out at the same time of the day.

### Statistical analysis

Results were expressed as mean ± standard deviation (mean ± SD), while the Students't test was used to determine the difference between means. P values less than 0.05 were taken as statistically significant. SPSS version 16 (Ill, Chicago) was used for the statistical analysis. Tables were used to display the results.

### Ethical review

The study was approved by the Ethical Committee of the University of Maiduguri Teaching Hospital.

## Results

A total of 255 medical students (169 males, 86 females) were enrolled in the study. The mean age of subjects was 24.8±3.2years (25.4±3.4 in males and 23.7±2.6 in females). The mean height of the subjects was 1.68±0.09m (1.7±0.08 in males and 1.6±0.06 in females). The males had a mean weight of 66.2kg and the females a mean weight of 57.9kg. Table 1 shows the age and anthropometric characteristics of the subjects. The youngest female was 18 years old and the youngest male 20 years old. The 23–27 year age group contributed the most in both sexes. None of the females was in the 33–37 years age group.

**Table 1:** Age, anthropometric characteristics and mean PEF of medical students

Parameter	Male	Female
**N**	**169**	**86**
Age	25.4±3.4	23.7±2.6
Weight (Kg)	66.2±10.6	57.9±12.5
Height (cm)	1.7±0.08	1.6±0.06
BMI (Kg/m^2^)	22.4±3.6	22.2±4.8
Waist circumference (cm)	78.9±8.5	73.9±9.3
PEF	518.8±99.2	369.2±60.4

BMI: Body mass index; PEF: Peak Expiratory flow

There was a statistically significant positive correlation between PEF and measured anthropometric variables. It was also positively correlated with age. Table 2 shows the correlation between PEF and the measured parameters.

**Table 2:** Correlation between age, anthropometric parameters and Peak expiratory flow

Parameter	Peak expiratory flow	
	*r*	*P*
Age (years)	0.18	0.004
Height (m)	0.43	0.000
Weight (Kg)	0.38	0.000
Waist circumference (cm)	0.26	0.000
BMI (Kg/m^2^)	0.15	0.016

BMI: Body Mass Index

Table 3 and Table 4 shows a comparison of the mean values derived from this sample of medical students and the predicted values derived from several population-based studies in Nigeria in females and males respectively.

**Table 3:** Comparison between measured and predicted values of PEF according to age groups in females

Age Range	n	Measured	Predicted			
			Njoku et al.	Nku et al.	Femi-Pierce et al.	Salisu et al.
18–22	25	355.2±60.9	414.4±16.1*	343.8±13.9**	360.6±21.4**	369.2±2.5**
23–27	55	374.4±58.2	426.04±17.5*	359.9±14.6**	363.9±26.2**	371.4±1.9**
28–32	6	380.0±78.2	409.4±15.3**	358.8±13.5**	332.9±22.4**	373.57±0.8**
33–37	0	NA	NA	NA	NA	NA

* P <0.05

** P>0.05

NA= Not available

**Table 4:** Comparison between measured and predicted values of PEF according to age groups in males

Age Range	n	Measured	Predicted			
			Njoku *et al*.,	*p*	Femi-Pierce *et al*.,	*p*
18–22	22	502. 3±106.2	532.4±40.3	0.22	439.99±42.09	0.014*
23–27	106	524.4±93.3	550.9±32.1	0.006*	445.69±33.68	0.000*
28–32	39	509.5±112.4	551.8±26.1	0.025*	434.38±27.52	0.000*
33–37	2	585.0±49.5	545.5±31.3	0.44	426.46±31.57	0.062

* Significant *p* value

## Discussion

The results of this study have revealed some interesting relationships between the measured PEF and the predicted values based on different formulae derived in Nigeria. The differences may possibly be due to the fact that these formulae were got from certain tribes or clans whose cultures, diet and body build could be distinct [10]. The males had higher PEF consistent with all the studies done in Nigeria and elsewhere [1, 2, 4]. Males were heavier and taller than the females. These factors invariably affect PEF. The males in this study were also slightly older than the females.

There was a statistically significant positive correlation between PEF and all the measured anthropometric parameters in this study. This is in agreement with earlier studies done in Nigeria [8, 11].

The positive correlation of PEF with age was similar to that documented among females in south eastern Nigeria [13] and also with several other reports [8–10, 12, 13]. This was, however, contrary to the findings of a study done in Nigeria which reported a negative correlation of PEF with age. Their population included people >39 years of age and some studies have shown decline in PEF with age after 39years [14]. The age range in this study included a relatively few numbers of individuals 30 years and above and none above 39 years.

The measured PEF in this study were lower than those predicted by Njoku et al., [1]. It is, however, the one which provided the closest values to those measured among the males in this population. Their formula was derived using curvilinear formulae and seems robust. It, however, tends to estimate a relatively higher percentage PEF than the other formulae. Its advantages include the fact it accommodates the real trend of change in PEF with age.

The values in the females are most consistent with those predicted in a recent study done in North West Nigeria [15]. This is not surprising as the population they used was similar to that in this study in terms of region and cultural practices. Their equation also meets two of the three criteria recently suggested by some researchers [16].

Elebute and Femi-Pearse [4] formula tended to under-predict PEF especially in males. A similar observation was also made by some researchers [1]. Changes in secular trend were given as possible reasons for low values.

## Conclusion

The prediction formulae of Njoku et al. and Salisu et al. can be used in the assessment of individuals in this environment. This however, does not preclude a future larger sample size study.
